# Another Year to Celebrate Advances in Clinical Hematology

**DOI:** 10.1007/s44228-023-00040-y

**Published:** 2023-03-20

**Authors:** Mohamad Mohty, Junia V. Melo

**Affiliations:** 1 Service d’Hématologie Clinique et Thérapie Cellulaire, Sorbonne University, Saint-Antoine Hospital (AP-HP), and INSERM UMRs 938, Paris, France; 2 Faculty of Health and Medical Sciences, University of Adelaide, Adelaide, Australia

As we embark on the new year, 2023, it is a great opportunity to thank our authors, reviewers, contributors, supporters and readers for their commitment to the journal, and dedicated voluntary work on behalf of the International Academy for Clinical Hematology (IACH).

The past year was marked by notable progress in our scientific and educational output, academic offerings and new initiatives, while we continued to navigate an evolving state of the COVID-19 pandemic. Together, we are strengthening the foundations of our vision for “Hematology without Borders”®, and are now ready to plan for continuous and strong growth on vital fronts.

In 2022, we have launched the “Standing on the shoulders of the giants” ® initiative. This aims to celebrate the achievements of leading experts and investigators whose work and research have helped to significantly advance the field, and who established the milestones and foundations of modern clinical hematology. The IACH Scientific Steering Committee chooses the honorees from different fields of clinical hematology. All IACH members, friends and supporters are also encouraged to propose names. The steering committee will ultimately choose among the proposed names based on each nominee’s body of work, including strength of research, clinical impact, significant educational contributions, and overall accomplishments. The honoree will participate into a special webinar (interview format), where his/her landmark successes will be described and discussed, while underlining the remarkable character of these achievements and their tremendous impact in clinical practice. The transcript of the interview will be published in this journal [1, 2].

Another key initiative was the “IACH Junior Club” which aims to attract the younger generation of new investigators into the field and prepare for the future. The Club is bringing together young colleagues from across the globe to collaborate, share experiences and, most importantly, put a special focus on the “patient-first” approach through discussion of real-life clinical cases. The goal of the IACH Junior Club initiative is to spotlight the work that our younger colleagues, students, residents, fellows, registrars, assistants, etc., are doing every day to help those living with blood diseases. The most complex clinical cases are presented by the younger colleagues and discussed with top experts in the field (https://clinical-hematology.org/junior-club).

Last, but not least, we have kicked-off “The Innovation and R&D Podium” of the IACH. This activity aims to establish a dialogue between pharmaceutical companies and academia: the company is invited by the IACH to present its pipeline and objectives, and interact with academic experts and the audience about the pipeline, ongoing research and future perspectives. The idea is to raise awareness of the ongoing progress, spread the news, and inform the professionals about “What’s coming next?”. Beside its scientific value, such high-level dialogue and dissemination is extremely important to bring all stakeholders together and accelerate the pace of innovation in clinical hematology (https://clinical-hematology.org/rd-podium).

The IACH meetings’ portfolio is also steadily growing, with the recent introduction of specific and specialized tracks dedicated to myelodysplastic syndromes and acute leukemia, lymphoma and chronic lymphoid disorders, and myeloproliferative neoplasms.

Our priorities and guiding principles in 2023 and subsequent few years will be focused on keeping a close eye on the rapidly changing landscape of clinical hematology education, and living by our mission and values. As the field becomes increasingly diverse and complex, how do we ensure inclusion of all topics, and what pathways and tools should we design to enable their realization and success?

Besides top-notch open-access education, the other important major project we are working on is to push the IACH into the clinical research arena, in order to scale and steer a research enterprise towards some relevant and impactful projects for the benefit of patients (whether prospective, retrospective or registry-based clinical research). Building such an ambitious infrastructure would require a lot of effort, hard work and resources, but would definitely encourage and facilitate international research collaboration across the IACH community. The IACH is committed to mobilizing resources for leadership in the creation and translation of knowledge, and towards innovation in the development of clinical research and interventions that shape the well-being of hematology patients. Creative work and academia-driven research questions will be our priority to meet our vision where decisions about clinical hematology practice and care are informed by high-quality evidence. In the coming months, we will work on those priorities, and welcome your thoughts and suggestions in the exchange of ideas and insights around them.

With your help and support for over 5 years, the IACH has been building on the dedication of expert volunteers to create new ways for sharing knowledge, and for producing the right guidance and practical recommendation, in a timely manner, to support decision making. Our results demonstrate an unprecedented scale of progress, with the IACH becoming the most popular and most credible one-stop shop for digital education. There is still much to do but, for sure, the IACH is committed to be always the leader in innovation, and will pioneer some transformative groundbreaking changes. As we look towards new frontiers, we will to continue to collaborate and work with the global clinical hematology community to create more impact. This new issue marks the fourth anniversary of the journal Clinical Hematology International. [3] You will read some high-level clinical reviews and original reports. In this era of hematology revolution, we hope you will continue to enjoy the IACH activities.

## Data Availability

Not applicable.
